# Toward an Understanding of the Molecular Mechanisms of Barnacle Larval Settlement: A Comparative Transcriptomic Approach

**DOI:** 10.1371/journal.pone.0022913

**Published:** 2011-07-29

**Authors:** Zhang-Fan Chen, Kiyotaka Matsumura, Hao Wang, Shawn M. Arellano, Xingcheng Yan, Intikhab Alam, John A. C. Archer, Vladimir B. Bajic, Pei-Yuan Qian

**Affiliations:** 1 KAUST Global Collaborative Research Program, Division of Life Science, The Hong Kong University of Science and Technology, Hong Kong SAR, China; 2 Woods Hole Oceanographic Institution, Woods Hole, Massachusetts, United States of America; 3 Red Sea Laboratory for Integrative Systems Biology, King Abdullah University of Science and Technology, Thuwal, Kingdom of Saudi Arabia; Institute of Marine Research, Norway

## Abstract

**Background:**

The barnacle *Balanus amphitrite* is a globally distributed biofouler and a model species in intertidal ecology and larval settlement studies. However, a lack of genomic information has hindered the comprehensive elucidation of the molecular mechanisms coordinating its larval settlement. The pyrosequencing-based transcriptomic approach is thought to be useful to identify key molecular changes during larval settlement.

**Methodology and Principal Findings:**

Using 454 pyrosequencing, we collected totally 630,845 reads including 215,308 from the larval stages and 415,537 from the adults; 23,451 contigs were generated while 77,785 remained as singletons. We annotated 31,720 of the 92,322 predicted open reading frames, which matched hits in the NCBI NR database, and identified 7,954 putative genes that were differentially expressed between the larval and adult stages. Of these, several genes were further characterized with quantitative real-time PCR and *in situ* hybridization, revealing some key findings: 1) vitellogenin was uniquely expressed in late nauplius stage, suggesting it may be an energy source for the subsequent non-feeding cyprid stage; 2) the locations of mannose receptors suggested they may be involved in the sensory system of cyprids; 3) 20 kDa-cement protein homologues were expressed in the cyprid cement gland and probably function during attachment; and 4) receptor tyrosine kinases were expressed higher in cyprid stage and may be involved in signal perception during larval settlement.

**Conclusions:**

Our results provide not only the basis of several new hypotheses about gene functions during larval settlement, but also the availability of this large transcriptome dataset in *B. amphitrite* for further exploration of larval settlement and developmental pathways in this important marine species.

## Introduction

Barnacles are one of the most dominant sessile organisms in marine intertidal communities. In particular, the striped barnacle *Balanus ( = Amphibalanus) amphitrite* is distributed worldwide [1] and is the predominant biofouler in the world's ports [2]. Due to the tremendous global economic losses to maritime industries caused by biofouling, considerable efforts have been made to develop suitable antifouling technology. The attachment and metamorphosis (collectively known as “larval settlement”) of barnacle larvae is a crucial process by which barnacles permanently adhere to surfaces; larval settlement, therefore, has been the basic target of understanding for biofouling and antifouling studies, in addition to ecological studies.

Gregarious settlement by barnacle larvae is believed to be based on the transduction of chemical signals from conspecifics [3], [4]. A novel glycoprotein that induces gregarious settlement, settlement-inducing protein complex (SIPC), has been isolated, characterized, and sequenced from adult *B. amphitrite* barnacles [5], [6]. SIPC is expressed in cyprids and their footprints deposited on the surface during exploration behavior [7], [8], suggesting that it is involved in chemical communication among larvae and between larvae and adults during settlement. Although the relevant SIPC receptors in cyprids remain unknown, signal transduction during cyprid settlement has been explored by examining the effects of various compounds on barnacle larval settlement [4]. The results suggest that G protein-linked receptors, cyclic AMP, calcium ions, and neurotransmitters are all involved in the signal transduction of cues for larval attachment and cement secretion [9]–[11]. Metamorphosis, on the other hand, seems to be controlled by hormones such as 20-hydroxyecdysone (20-HE) and methyl farnesoate, which may regulate protein kinase C activation [12]–[17]. Although these previous studies have contributed to our understanding of cyprid settlement mechanisms, most provide only indirect evidence of the signal transduction pathways and are far from a systematic understanding of the molecular network involved in barnacle larval settlement.

More recently, molecular biology approaches have revealed some new insights into the gene and protein expression changes that occur during barnacle larval settlement [18]. Proteome and phosphoproteome analyses of *B. amphitrite* showed dramatic changes in protein expression profiles during larval development and metamorphosis [19], and distinct phosphoproteome patterns associated with different developmental stages suggested that larval settlement is mediated by protein phosphorylation status [20]. Furthermore, it was suggested that certain proteins involved in stress regulation and energy metabolism play crucial roles in regulating larval attachment and metamorphosis of *B. amphitrite* [21]. Using cDNA libraries and Northern blot analysis, Okazaki and Shizuri [22] identified six genes (*bcs1-6*) that were specifically expressed in *B. amphitrite* cyprids and found that the expression levels of these genes changed after exposing cyprids to artificial inducing and inhibitory cues [23]. The expression profiles of the genes *bcs1-6* in *B. amphitrite* differed at different developmental stages, suggesting that they play different roles during settlement [24], but their specific functions have not been clarified. In addition, several receptor genes in barnacles that might be involved in signal transduction during cyprid settlement have been cloned and sequenced [25]–[27]. Finally, De Gregoris et al. [28] constructed a cDNA library for adult *B. amphitrite* and analyzed the expression levels of selected genes, identifying several genes that might be useful for future studies of the molecular mechanisms of barnacle larval settlement.

The construction of cDNA libraries with the Sanger sequencing method is time consuming and frequently yields insufficient coverage of the transcriptome, though. In comparison, the recently developed 454-pyrosequencing technology can rapidly generate a large number of reads and has several advantages over the Sanger method [29]–[31]. Consequently, it has become a powerful platform for profiling the transcriptomes of both model and non-model organisms [32], [33], [34]. In this study, we applied 454-pyrosequencing technology to compare the transcriptomes of *B. amphitrite* larvae and adults. We also examined the expression profiles during larval development and settlement of selected genes to assess whether they are involved in larval settlement mechanisms. Finally, we use our findings to suggest new directions in the study of larval settlement that might reveal the molecular network of this crucial process in marine sessile organisms.

## Results and Discussion

### Transcriptome Profiling of the Barnacle *Balanus amphitrite*

**Figure 1:**
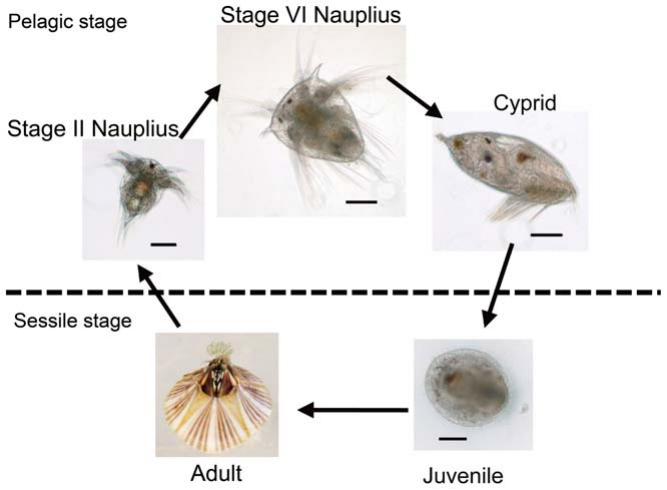
Developmental stages of the barnacle *Balanus amphitrite* that were sampled for transcriptomic and gene expression analysis. The stages include stage II nauplius, stage VI nauplius, cyprid, juvenile and adult. The larval cDNA pool consisted of stage II nauplius, stage VI nauplius and cyprid, while the adult cDNA pool was staged only. The diameter of the adult barnacle is ∼2 cm. Scale bars  = 100 µm.

**Figure 2:**
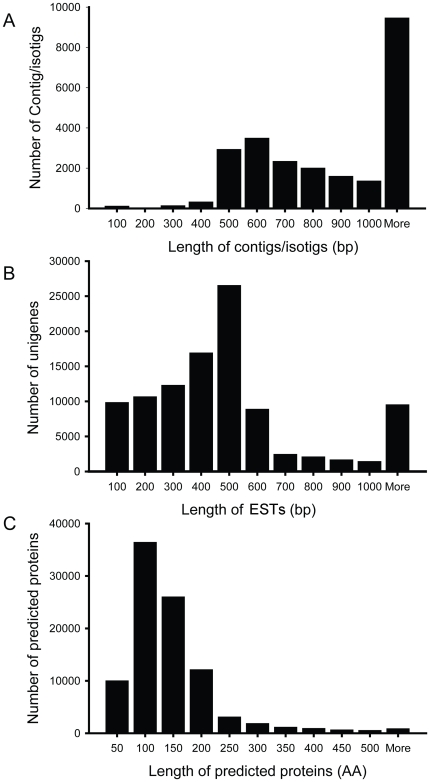
The size distributions of the contig/isotigs (A), the total ESTs (B), and the deduced open reading frames (ORFs) (C).

To cover the transcriptome of different developmental stages of *B. amphitrite*, two cDNA pools were prepared: a larval cDNA pool, which contained cDNA from three larval stages (Figure 1), and an adult cDNA pool. The 454 pyrosequencing was performed on both cDNA pools independently. The results of the pyrosequencing are summarized in Table 1. A total of 226 Mbp of transcriptome data were generated from the *B. amphitrite* 454 pyrosequencing, comprising 630,845 confident reads with an average length of 358 bp. The raw sequencing data was submitted to the Short Read Archive (SRA) of NCBI (Accession number: SRA029164.1). All of the reads were pooled together for *de novo* transcriptome assembly using Newbler 2.3 software, which sorts the reads into contigs (ideally, aligned reads from a single transcript), isotigs (splice variants of contigs), and isogroups (groups of isotigs from a single gene). The sequences were assembled into 23,451 contiguous sequences (182 contigs and 23,269 isotigs) with an average length of 1,116 bp, and 77,785 remained as singletons. In total, 101,236 ESTs (expressed sequence tag) with an average length of 502 bp were generated from the *B. amphitrite* transcriptome profiling. The size distributions of the contiguous sequences and the ESTs are shown in Figure 2.

**Table 1:** Summary of the barnacle *B. amphitrite* transcriptome.

**Total number of qualified reads**	630,845
**Qualified reads from larval pool**	215,308
**Qualified reads from adult pool**	415,537
**Average read length**	358 bp
**Total number of contig/isotigs**	23,451
**Mean length of contig/isotigs**	1,116 bp
**Total number of ESTs**	101,236
**Average length of ESTs**	502 bp
**Total number of predicted open reading frames (ORFs)**	92,322
**Average length of ORFs**	358 bp

**Figure 3:**
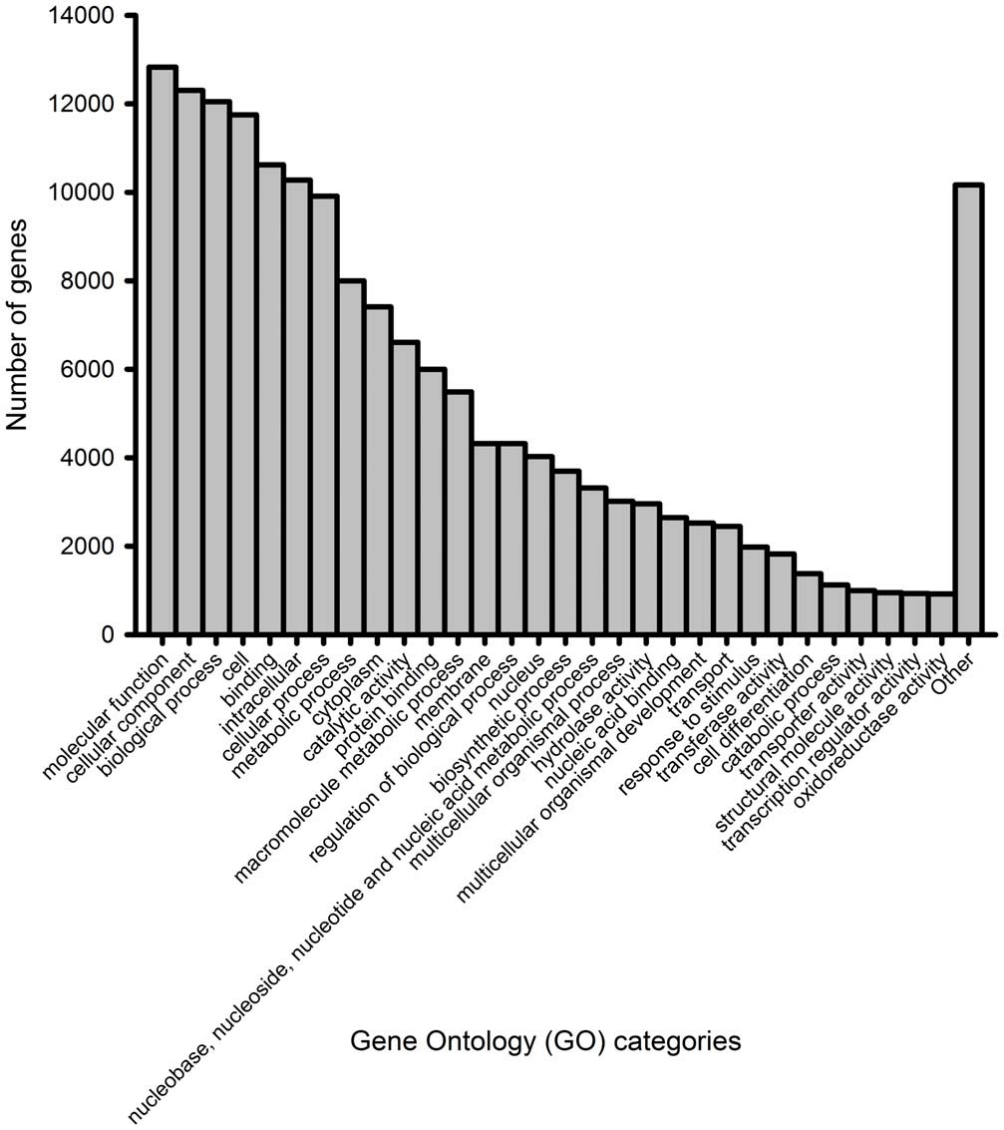
Top 30 gene ontology (GO) categories of *B. amphitrite* transcriptome.

A total of 92,321 open reading frames (ORFs) were predicted, with 31,720 ORFs that matched to the NCBI NR database. The size distribution of ORFs is shown in Figure 2C. The remaining 60,601 were unmatched and were annotated as hypothetical proteins. To give an overview of all the different functional classes in the barnacle transcriptome database, the sequences were also annotated with gene ontology (GO) terms. Based on sequence homology, 166,803 sequences can be categorized into 62 functional groups. The top 30 most abundant groups were shown in Figure 3.

### Comparative Transcriptomic Analysis

**Figure 4:**
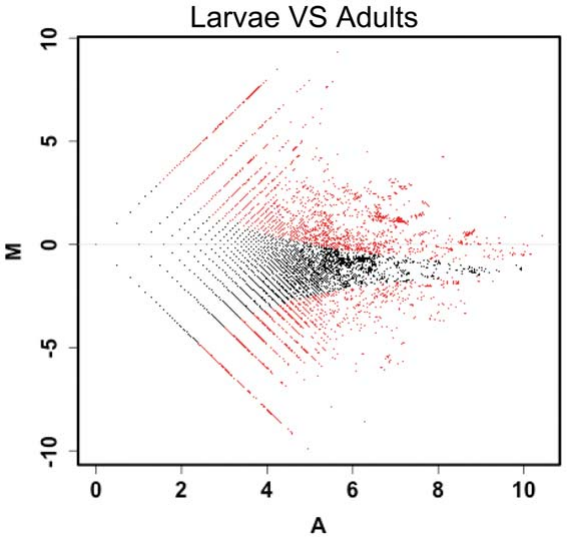
The MA-plot generated by the DEGseq gene expression profile analysis. The M is the reads number ratio and the A is the average. The red dots indicate the differently expressed genes between the larval and adult cDNA pools.

Because *B. amphitrite* larvae are morphologically distinct and occupy a different ecological niche from adult barnacles, we hypothesized that genes that were more highly expressed in the larval cDNA pool than in the adult cDNA pool were likely to play roles in larval development and settlement. The DEGseq analysis of contig/isotig reads numbers revealed that there were in total 7,954 contigs/isotigs were differentially expressed between the larval and adult pools (p<0.001; Figure 4). Among these, 743 contigs/isotigs were uniquely expressed in the larval stages; 443 were expressed at least 10 folds higher in the larvae than in the adults; and 1,318 were at least two folds higher in the larvae than in the adults (Table S1). On the other hand, 2,534 contigs/isotigs were exclusively expressed in the adult stage, 1,240 were at least 10 folds higher in the adult stage, and 546 were at least 2 folds higher in the adults than in the larvae.

**Figure 5:**
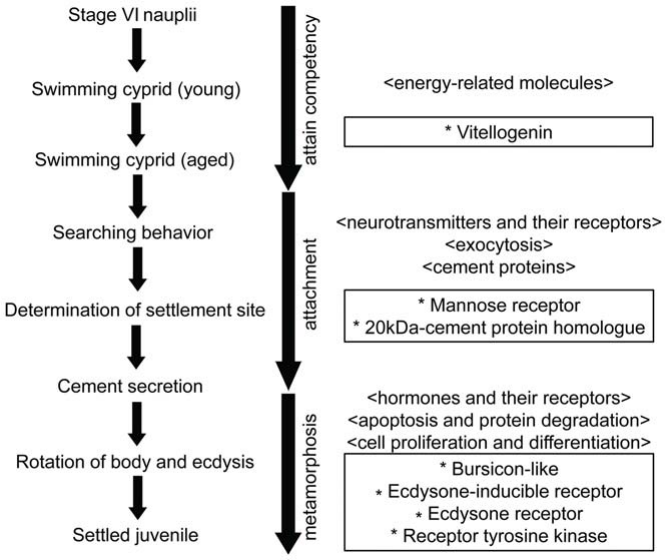
Phases of the larval settlement process in *B. amphitrite*. Functional gene groups and genes that were differentially expressed and may be involved in this process are indicated in Table S2.

The larval settlement process of barnacles is thought to comprise three steps: larvae must first attain competency to settle, then attach on the substratum, and finally metamorphose into juveniles (Figure 5, Table S2). Details of the attainment of competency have not been understood well, but energy-related molecules and physiological changes during early cyprid development may be involved in this process [21], [35]. Using a proteomic analysis, Zhang et al. [21] identified several stress regulation and energy metabolism proteins that were purportedly involved in development of *B. amphitrite* cyprids [21]. Similarly, transcripts for heat shock proteins were more highly expressed in the larval stages than the adults (Table S2). After swimming for several hours and attaining competency, cyprids change their behaviors and begin searching the substratum, finally selecting a settlement site [36], [37]. Cyprids have stage-specific compound eyes and it has been suggested cyprid photo-reception is involved in larval settlement behavior [38]. Here, we found several photoreceptor-related genes that were highly expressed in the larval stages and significantly down-regulated in the adults (Table S2). During attachment, the cyprid secretes cement proteins to glue itself onto the substratum. In this second step, neurotransmitter molecules such as dopamine and serotonin, their receptors, and cement proteins may be involved [39]–[41]. Metamorphosis in barnacles is thus believed to be controlled by some hormones like methyl farnesoate and their receptors [15], [16]. Indeed, we found a suite of hormone receptors that were more highly expressed in the larval stages than in the adults (Table S2). Additionally, because metamorphosis of barnacles entails considerable morphological rearrangement, this final step in larval settlement may also include apoptosis, controlled protein degradation, cell-cell interactions, cell proliferation, and cell differentiation. Many genes in these functional groups were identified and differentially expressed in the barnacle transcriptomes (Table S2). Finally, signal reception and signal transduction should be important during many phases of barnacle settlement. Barnacle cyprids are known to respond behaviorally to various chemical and tactile cues, ultimately culminating in habitat selection, attachment, and metamorphosis; all of which should involve signal reception and transduction. Here, we identified differentially regulated genes belonging to several important signaling pathways, including receptor tyrosine kinases, hormone receptors, Wnt-signaling related genes and so on (Table S2).

### Gene Expression Profiling

Of the differentially expressed genes (Table S2), vitellogenin, mannose receptors, 20 kDa-cement protein homologues, the receptor tyrosine kinase family, hormone receptors, and cubilins were selected for comparison of their expression profiles in different developmental stages by using real-time PCR.

**Figure 6:**
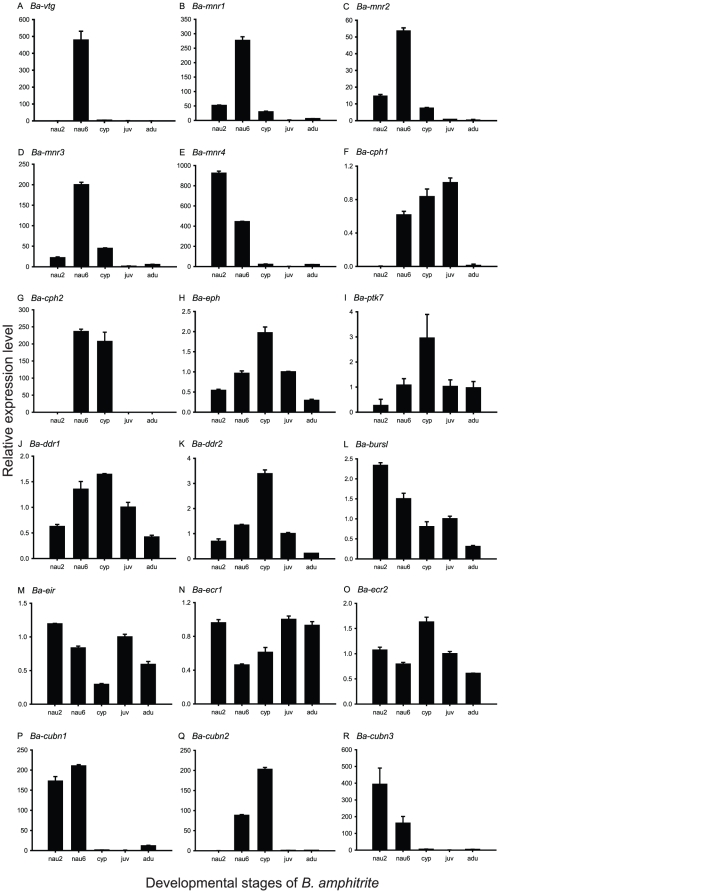
Real-time PCR results for the expression of 18 genes in five different developmental stages. The stages include the stage II nauplius (nau2), the stage VI nauplius (nau6), the cyprid (cyp), the young juvenile (juv) and the adult (adu). Expression was measured for (A) *Ba-vtg*, vitellogenin; (B) *Ba-mnr1*, mannose receptor 1; (C) *Ba-mnr2*, mannose receptor 2; (D) *Ba-mnr3*, mannose receptor 3; (E) *Ba-mnr4*, mannose receptor 4; (F) *Ba-cph1*, 20 kDa-cement protein homologue 1; (G) *Ba-cph2*, 20 kDa-cement protein homologue 2; (H) *Ba-eph*, epidermal growth factor receptor; (I) *Ba-ptk7*, tyrosine-protein kinase-like 7; (J) *Ba-ddr1*, discoidin domain receptor 1; (K) *Ba-ddr2*, discoidin domain receptor 2; (L) *Ba-bursl*, bursicon-like; (M) *Ba-eir*, ecdysone-inducible protein; (N) *Ba-ecr1*, ecdysone receptor 1; (O) *Ba-ecr2*, ecdysone receptor 2; (P) *Ba-cubn1*, cubilin 1; (Q) *Ba-cubn2*, cubilin 2; and (R) *Ba-cubn3*, cubilin 3. Values are expressed as mean ± SD from three different experimental replicates.

Vitellogenin is an egg yolk precursor protein that is related to lipid transport [42]. It is synthesized extraovarially in females and deposited in the developing oocytes, generating vitellin, the main yolk protein [43]. Vitellin is consumed as a nutrient during embryogenesis. Vitellogenin and vitellin have been studied extensively as energy sources in the vitellogenesis of fish [44], [45], insects [46], [47], crustaceans [48], [49], and polychaetes [50]. Although vitellin is generally found as an egg yolk protein expressed in females, a vitellin-like protein has been uniquely found in barnacle cyprid larvae [35]. For barnacle larvae, lipids and proteins are the primary energy sources, especially for non-feeding cyprids, and are believed to be involved in exploration and attainment of competency by swimming larvae [51]. In *B. amphitrite*, a vitellin-like protein was found to accumulate in the late nauplius stage and reached a peak at the cyprid stage, then decreased greatly during metamorphosis, suggesting this protein provided energy for the non-feeding cyprid [35]. Our real-time PCR results showed the most prominent gene expression of vitellogenin (*Ba-vtg*) in the late nauplius stage (Figure 6A), suggesting that there is a time lag between transcription of barnacle vitellogenin and peak expression of vitellin-like protein during the cyprid stage [35]. The barnacle larvae may need to accumulate the vitellogenin transcripts before development to the competent cyprid stage.

**Figure 7:**
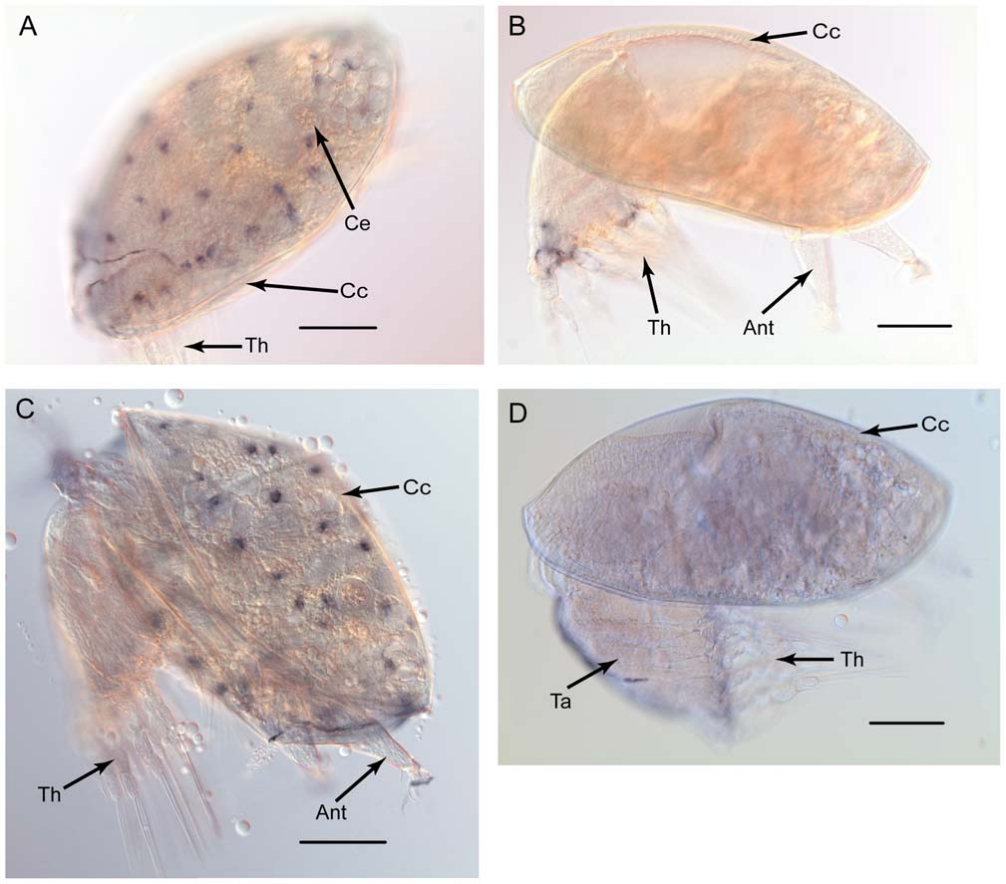
Gene spatial expression patterns of *B. amphitrite* mannose receptor genes, *Ba-mnr1* (A, B) and *Ba-mnr2* (C, D). (A) lateral view of a cyprid; (B) negative control; (C) lateral view of a cyprid; (D) negative control. Abbreviations: Ant, antennule; Cc, cyprid bivalve carapace; Ce, compound eye; Ta, thorax; Th, thoracopod. Scale bars  = 100 µm.

Similar gene expression patterns were observed in mannose receptors. The settlement-inducing protein complex (SIPC), a kind of glycoprotein, is reportedly a biological cue for the gregarious settlement of *B. amphitrite* [6]. Lentil lectin (LCA), a mannose-binding lectin, binds to SIPC and inhibits SIPC-induced larval settlement [5], [52]. These results suggest that SIPC contains mannose-type sugar chains, and mannose receptor-like proteins in cyprids may be involved in the recognition of SIPC. LCA also inhibits the periphytic diatom-induced larval settlement of *B. amphitrite* [53], indicating that mannose receptors may also be related to biofilm recognition. We found several isotigs annotated as “mannose receptors” that were highly expressed in the larval transcriptome, and selected four isotigs for further expression profile analysis. These four mannose receptor genes were highly expressed in stage VI nauplii, and expression of mannose receptors 1 to 3 were especially high in this stage (Figure 6B–D). To detect the spatial pattern of the mannose receptors, *in situ* hybridization was performed on mannose receptors 1 and 2 in cyprids (Figure 7). Because SIPC is a surface-bound pheromone that is thought to be recognized during cyprid substratum searching behavior, it has been suggested that SIPC receptors may be located on the antennules [4]. However, we could not clearly detect the gene expression of the selected mannose receptors on the antennules. It is possible that the mannose receptors reported here are kinds of mechanoreceptors, which are different from the real SIPC receptor. Therefore, whether mannose receptors relate to SIPC remains unclear at this stage. However, we indeed found the mannose receptor expression associated with other putative sensory structures though. Cyprids of *B. amphitrite* carry pores and setae on their carapace [54] that are the putative sensory structures [55]. Interestingly, the mannose receptor genes were expressed in discrete areas of the surface of the cyprid carapace (Figure 7), which coincides with the locations of these pores and setae [54]. Although this suggests a possible relationship between mannose receptors and the sensory system in barnacle cyprids, the real functions of the pores and setae remain unclear.

**Figure 8:**
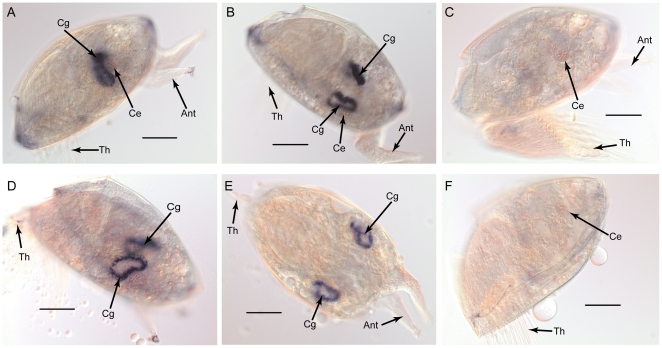
Gene spatial expression pattern of *B. amphitrite* 20-kDa cement protein homologue genes, *Ba-cph1* (A, B, C) and *Ba-cph2* (D, E, F). (A) lateral view of a cyprid; (B) dorsal-lateral view of a cyprid; (C) negative control; (D) lateral view of a cyprid; (E) dorsal view of a cyprid; (F) negative control. Abbreviations: Ant, antennule; Ce, compound eye; Cg, cement gland; Th, thoracopod. Scale bars  = 100 µm.

During barnacle larval settlement, cement proteins are secreted to attach cyprids to the substratum. There are two kinds of cement proteins, a primary cement protein that is produced while the barnacle is attaching to the substratum and the secondary cement protein that is secreted to aid barnacle's reattachment [56]. Several studies have examined both primary and secondary cement proteins in adult *Megabalanus rosa* barnacles [57], [58]. We found some isotigs annotated as barnacle cement proteins or their homologues in our transcriptome dataset. Some of these, such as cement protein-100k were only found in the adult transcriptome. However, some 20 kDa-cement protein homologues were highly expressed in the larvae (higher than in the adults). We analyzed the gene expression profiles of the 20 kDa-cement protein homologues by using real-time PCR. The 20 kDa-cement protein homologue gene 1 transcript (*Ba-cph1*) increased with larval development and settlement, but decreased in the adult adults. The 20 kDa-cement protein homologue gene 2 transcript (*Ba-cph2*) was expressed lower in stage II nauplii, young juveniles, and adults, but significantly higher in stage VI nauplii and cyprids (Figure 6F, G). *In situ* hybridization was carried out to confirm the spatial gene expression. Figure 8 shows that both genes of the 20 kDa-cement protein homologues were expressed in the pairs of cement glands. This implies that these two genes are involved in cement secretion during larval settlement in barnacles. In studies of barnacle cementation, adult cement proteins have been studied well [57], [58], but few reports have been published on larval cement proteins. Our results suggest that some cement proteins are predominantly expressed in larvae, but may have similar structure to adult cement proteins.

The receptor tyrosine kinase family contains 17 subfamilies, 13 of which can be found in the barnacle transcriptome dataset. This receptor family includes receptors for growth factors, differentiation factors, and factors that stimulate metabolic responses [59]. The insect prothoracicotropic hormone (PTTH) has been reported to activate “Torso,” a receptor tyrosine kinase that regulates embryonic terminal cell fate to initiate metamorphosis in *Drosophila* [60]. We used real-time PCR to analyze the expression pattern of several receptor tyrosine kinases that appeared to be differentially expressed between the larvae and adult based on transcript read numbers. The results showed that all of the genes were up-regulated in cyprids (Figure 6H–K). The gene expression patterns were similar for Eph receptor tyrosine kinase (*Ba-eph*), discoidin domain receptors (*Ba-ddr1* & *2*), and tyrosine protein kinase-like 7 (*Ba-ptk7*); they all increased with larval development, reaching highest expression in the cyprid stage, and then decreased after settlement. These results suggest that these tyrosine kinases play a role in barnacle larval settlement.

Besides the receptor tyrosine kinase family, some hormones and their receptors are well known to regulate in the metamorphosis of insects [61] and may play a role in barnacle metamorphosis as well [15], [16]. We selected certain known metamorphosis- or molting-related hormones and their receptors that had higher expression levels in the larval transcriptome dataset, and examined them further using real-time PCR. First, bursicon, the ultimate hormone in insect ecdysis that is involved in cuticle hardening, was reported to be expressed in certain identified neurons in the nervous systems of crustaceans as well [62]. In the *B. amphitrite* in this study, a bursicon-like gene (*Ba-bursl*) was expressed at its highest level in the stage II nauplii. Its expression then decreased during larval development. After settlement, the gene was slightly up-regulated in young juveniles and finally down-regulated in adults (Figure 6L). Second, ecdysone is a steroidal prohormone of the major insect molting hormone 20-HE. In addition to acting as a molting hormone in arthropods, ecdysone also occurs in other related phyla, but plays different roles. Figure 6M shows that the ecdysone-inducilble protein-related gene (*Ba-eir*) was expressed lowest in the cyprid stage compared to other stages. Additionally, the expression of ecdysone receptor gene 1 (*Ba-ecr1*) was also relatively low in the cyprids, but in contrast ecdysone receptor gene 2 (*Ba-ecr2*) was most abundantly expressed in the cyprids (Figure 6N, O). These results suggest that bursicon and ecdysone in *B. amphitrite* are not specifically involved in larval metamorphosis from cyprid to juvenile, but may be involved in molting during larval development. Furthermore, the insect juvenile hormone analogue methyl farnesoate (MF) reportedly induces larval metamorphosis and has previously been identified in cyprid larvae, suggesting that it plays an important role in the metamorphosis of barnacles [15], [16]. However, we did not find any MF or juvenile hormone-related genes in our transcriptome dataset. It is possible that some metamorphosis hormone-related genes in barnacles were included among the hypothetical proteins in our transcriptome.

Interestingly, the number of reads of cubilins, the vitamin B12 receptor, was significantly different between the larval and adult transcriptomes. However, the function of this gene remains unknown in most crustaceans. There were three cubilin genes represented in the barnacle transcriptome dataset, each of which showed a different expression profile. The cubilin 2 gene (*Ba-cubn2*) had 200 folds and 90 folds higher expression level in the cyprids and stage VI nauplii respectively than in the juveniles (Figure 6Q). The other two cubilin genes were both expressed at much higher levels in all of the naupliar stages, although they were differently expressed in the stage II nauplii and stage VI nauplii (Figure 6P, R). These interesting results implies that the three cubulin genes may play different roles during larval development, and that cubilin 1 may be particularly important for larval settlement. It should be pointed out that the most similar sequence to “cubilin 1” isotig in NCBI database indicated CUB-serine protease from the spiny lobster *Panulirus argus*. Interestingly, this CUB-serine protease was shown to be expressed in olfactory sensilla and the outer dendrites of olfactory receptor neurons, suggesting a possible function of this protease in the olfactory system [63]. In barnacle cyprids, an olfactory receptor neuron-like structure was reported in the fourth segment of the antennules [64] and olfactory chemoreception system has been suggested to be involved in larval settlement [38]. It is possible that cubilin 1, CUB-serine protease, in barnacle transcriptome is involved in olfactory-like chemoreception during barnacle larval settlement.

### Conclusions

We found several genes and functional groups that were differentially expressed and possibly involved in larval settlement in the barnacle transcriptome dataset. Importantly, we have provided support for several existing hypotheses about larval settlement in barnacles, as well as suggested new hypotheses that should be further explored. Our results suggest that several changes happen during late nauplius VI stage that may be important for the attainment of competency during the cyprid stage. First, the barnacle vitellogenin was uniquely expressed in the late nauplius stage and these transcripts may need to accumulate prior to development to the cyprid stage. Second, the peak expression of mannose receptors genes is in the stage VI nauplii and their localization patterns in cyprids suggest they may be involved in chemical or mechanical sensing during larval settlement. Third, 20-kDa cement protein homologues, which were previously unknown in barnacle larvae and may function during cyprid attachment, are highly expressed in both nauplii VI and in cyprid cement glands. Finally, we showed that receptor tyrosine kinase genes were strongly expressed in the cyprid stage. While the functions of some genes in this family have been suggested in other organisms, little has been reported on them in barnacles. We suggest this gene family may play an important role in larval settlement and are possibly involved in metamorphosis signal perception. Although the elucidation of the real biological functions of all these genes will require development of gene manipulation techniques such as RNAi for barnacles, we are hopeful that with the accumulation of new genomic data for barnacles these techniques will become possible in the near future.

Further studies on the molecular mechanisms of larval settlement will take advantage of our barnacle transcriptomic profiling, since the availability of a large transcriptome dataset for multiple life history stages of *B. amphitrite* will allow us to explore not only larval settlement in this important marine species but also other important pathways such as those involved in development, biomineralization, or stress regulation, for example.

## Materials and Methods

### Animal collection

Adults of *Balanus amphitrite* were collected from Pak Sha Wan, Hong Kong (22.21′45″ N, 114.15′35″ E). Larval collection and culture were performed according to Thiyagarajan and Qian [19]. For the 454 pyrosequencing, stage II nauplii, stage VI nauplii, cyprids, and adults samples were collected. For the real-time-PCR gene expression profile analysis, stage II nauplii, stage VI nauplii, cyprids, recently metamorphosed juveniles, and adults were collected. All of the samples were stored in liquid nitrogen until use.

### RNA Extraction and cDNA Synthesis

Total RNA was extracted with Trizol reagent (Invitrogen) according to the manufacturer's protocol. The total RNA was then treated with a Turbo DNA free kit (Ambion) to remove trace amounts of DNA contamination. For 454 pyrosequencing, messenger RNA was then purified from the total RNA with a PolyA Mag kit (Ambion). The double-stranded cDNA was synthesized with a random hexamer primer (Takara) and a Superscript double-strand cDNA synthesis kit (Invitrogen). Two cDNA pools were prepared for the titanium 454 pyrosequencing; one contained cDNA from three larval stages, including nauplius II (25% of the total cDNA), nauplius VI (25% of the total cDNA), and cyprids (50% of the total cDNA), and the other cDNA pool contained cDNA from adults. For the real-time PCR gene expression profile analysis, cDNA was synthesized from total RNA by using M-MLV reverse transcriptase (Ambion) with oligodT priming.

### 454 pyrosequencing, gene assembly, annotation, and bioinformatics analysis

The sequencing library construction and 454 pyrosequencing were performed according to the standard Roche 454 GS-FLX pyrosequencer protocol. The qualified pyrosequencing reads were assembled by using the Newbler software 2.3 (Roche) with default parameter settings (40 bp overlap, 90% identity). The open reading frame (ORF) prediction and gene annotation were performed according to methods previously described [65]. Briefly, all the putative ORFs of each EST were predicted by using GetORF, a in-house developed software. The ORFs were then aligned with NCBI NR peptide database by using BLASTp and the ORF with the highest score was used to annotate the EST. For the ESTs that did not have hits in the database, the longest ORF will be preserved and annotated as “hypothetical protein”. Gene ontology (GO) for all the genes was also predicted. In addition, we used BLASTx to aligh our dataset with nine bacteria genomes in order to identify any potential contaminants or symbionts. The detailed methods and results for the putative bacterial gene identifications are shown in Text S1 and Table S3, respectively. Pyrosequencing, assembly, and annotation were conducted by Chinese National Human Genome Center at Shanghai.

For each contig or isotig, the number of reads from each stage was extracted from the raw assembly file (ACE file). Differentially expressed contigs or isotigs were identified statistically by using Fisher's exact test using the DEGseq package developed by Wang et al. [66] for the R software environment (version 2.11.1 for Mac).

### Real-time PCR analysis

The primers used for the real-time-PCR analysis are listed in Table S4. The gene *cyb* was used as an internal control [28]. The real-time-PCR reactions were performed by using a KAPA SYBR fast universal 2X qPCR master mix on an ABI 7500 fast real-time PCR machine according to the standard protocol. The data analysis and statistical analysis were performed by using the relative quantitation (RQ) ΔΔCt method described by Livak and Schmittgen [67].

### *In situ* hybridization

The *B. amphitrite* cyprids were collected, relaxed in the mixture of 0.37M MgCl_2_ and autoclaved filtered seawater (1∶1) for 10 minutes, and then fixed with freshly prepared sample fixative solution (3.7% formalin in autoclaved filtered seawater) at 4°C overnight. The specimens were washed with PBST (1×PBS, 0.1% Tween 20, pH 7.4) three times, dehydrated with absolute methanol and stored in methanol at -20°C until use. The presence of the specific mRNA was detected by Digoxigenin (DIG) labeled antisense and sense RNA probes (Roche Applied Science). All the primers used are listed in Table S5. Whole-mount *in situ* hybridization was performed according to the methods described by Thisse and Thisse [68] with modifications. Briefly, the specimens were rehydrated by a successive dilution of methanol in PBS (75% methanol, 50% methanol, 25% methanol) and then washed 5 times with PBST. The specimens were then sonicated for 5 seconds. The samples were permeabilized with 10 µg/mL Proteinase K (Invitrogen) in PBST at room temperature for 7 minutes. The specimens were post-fixed in fixative solution (4% paraformaldehyde in PBS) and then washed 5 times with PBST. The specimens were pre-hybridized at 56°C for 2–4 hours in hybridization mix (50% formamide, 5×SSC, 50 µg/mL heparin, 1% SDS, 0.1% Tween 20, 500 µg/mL salmon sperm DNA). The hybridization was performed in 200 µL fresh hybridization mix containing 30–50 ng Dig-labeled RNA probe at 56°C overnight. Post-hybridization wash was performed with pre-warmed successively diluted hybridization mix (without heparin and salmon sperm DNA) in PBST (75%, 50%, and 25% hybridization mix) at 56°C and then washed 3 times with pre-warmed PBST. After cooling down to room temperature and 3 times additional washings with PBST, the specimens were blocked in blocking solution (2% sheep serum, 2% BSA in PBST) at room temperature for 2-4 hours and then incubated with 1/5,000 anti-DIG-AP (Roche applied science) in the blocking solution at 4°C overnight. The specimens were then washed 6 times with PBST at room temperature for 5 minutes each time and then incubated in Alkaline Tris buffer (100 mM Tris-HCl, pH 9.5, 50 mM MgCl_2_, 100 mM NaCl, 0.1% Tween 20) containing 4.5 mg/mL NBT and 3.5 mg/mL BCIP (Promega). The staining was performed at room temperature overnight in darkness, and then the specimens were washed several times with stop solution (1 mM EDTA, PBST). The specimens were mounted with 80% glycerol and observed under the microscope (BX-51 compound microscope, Olympus) with DIC setting and photographed.

## Supporting Information

Text S1 Method of putative bacterial gene identification.
(DOC)

Table S1 Important genes/pathways identified from transcriptome profiling.
(XLS)

Table S2 List of genes more highly expressed in the larval stages than the adults based on barnacle transcriptome.
(XLS)

Table S3 List of putative bacterial genes.
(XLS)

Table S4 List of primers for genes under real-time PCR assay.
(DOC)

Table S5 List of primers for genes under ISH study.
(DOC)

## References

[1] Darwin C (1854). A Monograph of the Sub-class Cirripedia, with Figures of all the Species.. The Balanidae (or Sessile Cirripedes); the Verrucidae, etc.

[2] Matias JR, Rabenhorst J, Mary A, Lorilla AA (2003). Marine biofouling testing of experimental marine paints: Technical considerations on methods, site selection and dynamic tests..

[3] Crisp DJ, Meadows PS (1962). The chemical basis of gregariousness in cirripedes.. Proc R Soc B.

[4] Clare AS, Matsumura K (2000). Nature and perception of barnacle settlement pheromones.. Biofouling.

[5] Matsumura K, Nagano M, Fusetani N (1998). Purification of a larval settlement-inducing protein complex (SIPC) of the barnacle, *Balanus amphitrite*.. J Exp Zool.

[6] Dreanno C, Matsumura K, Dohmae N, Takio K, Hirota H (2006). An alpha2-macroglobulin-like protein is the cue to gregarious settlement of the barnacle *Balanus amphitrite*.. Proc Natl Acad Sci U S A.

[7] Matsumura K, Nagano M, Kato-Yoshinaga Y, Yamazaki M, Clare AS (1998). Immunological studies on the settlement-inducing protein complex (SIPC) of the barnacle *Balanus amphitrite* and its possible involvement in larva-larva interactions.. Proc R Soc B.

[8] Dreanno C, Kirby RR, Clare AS (2006). Smelly feet are not always a bad thing: the relationship between cyprid footprint protein and the barnacle settlement pheromone.. Biol Lett.

[9] Rittschof D, Maki J, Mitchell R, Costlow JD (1986). Ion and neuropharmacological studies of barnacle settlement.. Netherlands J Sea Res.

[10] Clare AS, Thomas R, Rittschof D (1995). Evidence for the involvement of cyclic AMP in the pheromonal modulation of barnacle settlement.. J Exp Biol.

[11] Yamamoto H, Tachibana A, Kawaii S, Matsumura K, Fusetani N (1996). Serotonin involvement in larval settlement fo the barnacle, *Balanus amphitrite*.. J Exp Zool.

[12] Cheung PJ (1974). The effect of ecdysterone on cyprids of *Balanus eburneus* Gould.. J Exp Mar Biol Ecol.

[13] Freeman JA, Costlow JD (1983). The cyprid molt cycle and its hormonal control in the barnacle *Balanus amphitrite*.. J Crustacean Biol.

[14] Clare AS, Rittschof D, Costlow JD (1992). Effects of the nonsteroidal ecdysone mimic RH 5849 on larval crustaceans.. J Exp Zool.

[15] Yamamoto H, Okio T, Yoshimura E, Tachibana A, Shimizu K (1997). Methyl farnesoate induces larval metamorphosis of the barnacle, *Balanus amphitrite* via protein kinase C activation.. J Exp Zool.

[16] Smith PA, Clare AS, Rees HH, Prescott MC, Wainwright G (2000). Identification of methyl farnesoate in the cypris larva of the barnacle, *Balanus amphitrite*, and its role as a juvenile hormone.. Insect Biochem Mol Biol.

[17] Yamamoto H, Satuito CG, Yamazaki M, Natoyama K, Tachibana A (1998). Neurotransmitter blockers as antifoulants against planktonic larvae of the barnacle *Balanus amphitrite* and the mussel *Mytilus galloprovincialis*.. Biofouling.

[18] Thiyagarajan V (2010). A review on the role of chemical cues in habitat selection by barnacles: New insights from larval proteomics.. J Exp Mar Biol and Ecol.

[19] Thiyagarajan V, Qian PY (2008). Proteomic analysis of larvae during development, attachment, and metamorphosis in the fouling barnacle, *Balanus amphitrite*.. Proteomics.

[20] Thiyagarajan V, Wong T, Qian PY (2009). 2D Gel-based proteome and phosphoproteome analysis during larval metamorphosis in two major marine biofouling invertebrates.. J Prot Res.

[21] Zhang Y, Xu Y, Arellano SM, Xiao K, Qian PY (2010). Comparative proteome and phosphoproteome analyses during cyprid development of the barnacle *Balanus ( = Amphibalanus) amphitrite*.. J Prot Res.

[22] Okazaki Y, Shizuri Y (2000). Structures of six cDNAs expressed specifically at cypris larvae of barnacles, *Balanus amphitrite*.. Gene.

[23] Okazaki Y, Shizuri Y (2000). Effect of inducers and inhibitors on the expression of bcs genes involved in cypris larval attachment and metamorphosis of the barnacles *Balanus amphitrite*.. Int J Dev Biol.

[24] Li H, Thiyagarajan V, Qian PY (2010). Response of cyprid specific genes to natural settlement cues in the barnacle *Balanus ( =  Amphibalanus) amphitrite*.. J Exp Mar Biol Ecol.

[25] Isoai A, Kawahara H, Okazaki Y, Shizuri Y (1996). Molecular cloning of a new member of the putative G protein-coupled receptor gene from barnacle *Balanus amphitrite*.. Gene.

[26] Kawahara H, Isoai A, Shizuri Y (1997). Molecular cloning of a putative serotonin receptor gene from barnacle, *Balanus amphitrite*.. Gene.

[27] Lind U, Alm Rosenblad M, Hasselberg Frank L, Falkbring S, Brive L (2010). Octopamine receptors from the barnacle *Balanus improvisus* are activated by the alpha2-adrenoceptor agonist medetomidine.. Mol Pharmacol.

[28] De Gregoris TB, Borra M, Biffali E, Bekel T, Burgess JG (2009). Construction of an adult barnacle (*Balanus amphitrite*) cDNA library and selection of reference genes for quantitative RT-PCR studies.. BMC Mol Biol.

[29] Margulies M, Egholm M, Altman W, Attiya S, Bader J (2005). Genome sequencing in microfabricated high-density picolitre reactors.. Nature.

[30] Moore MJ, Dhingra A, Soltis PS, Shaw R, Farmerie WG (2006). Rapid and accurate pyrosequencing of angiosperm plastid genomes.. BMC Plant Biol.

[31] Weber AP, Weber KL, Carr K, Wilkerson C, Ohlrogge JB (2007). Sampling the Arabidopsis transcriptome with massively parallel pyrosequencing.. Plant Physiol.

[32] Wheat CW (2010). Rapidly developing functional genomics in ecological model systems via 454 transcriptome sequencing.. Genetica.

[33] Vera JC, Wheat CW, Fescemyer HW, Frilader MJ, Crawford DL (2008). Rapid transcriptome characterization for a nonmodel organism using 454 pyrosequencing.. Mol Ecol.

[34] De Gregoris TB, Rupp O, Klages S, Knaust F, Bekel T (2011). Deep sequencing of naupliar- cyprid- and adult-specific normalised Expressed Sequence Tag (EST) libraries of the acorn barnacle *Balanus amphitrite*.. Biofouling.

[35] Shimizu K, Satuito CG, Saikawa W, Fusetani N (1996). Larval storage protein of the barnacle, *Balanus amphitrite*: biochemical and immunological similarities to vitellin.. J Exp Zool.

[36] Crisp DJ, Costlow JD, Tipper RC (1984). Overview of research on marine invertebrate larvae.. Marine Biodeterioration: An Interdisciplinary Study.

[37] Walker G, Yule AB, Nott JA, Southward AJ (1987). Structure and function in balanomorph larvae.. Barnacle Biology.

[38] Anil AC, Khandeparker L, Desai DV, Baragi LV, Gaonkar CA (2010). Larval development, sensory mechanisms and physiological adaptations in acorn barnacles with special reference to *Balanus amphitrite*.. J Exp Mar Biol Ecol.

[39] Walker G (1971). A study of the cement apparatus of the cypris larva of the barnacle *Balanus balanoides*.. Mar Biol.

[40] Okano K, Shimizu K, Satuito C, Fusetani N (1996). Visualization of cement exocytosis in the cypris cement gland of the barnacle *Megabalanus rosa*.. J Exp Biol.

[41] Odling K, Albertsson C, Russell JT, Martensson LGE (2006). An *in vivo* study of exocytosis of cement proteins from barnacle *Balanus improvisus* (D.) cyprid larva.. J Exp Biol.

[42] Aderson TA, Levitt DG, Banaszak LJ (1998). The structural basis of lipid interactions in lipovitellin, a soluble lipoprotein.. Structure (London).

[43] Hagedorn HH, Kunkel JG (1979). Vitellogenin and vitellin in insects.. Ann Rev Entomol.

[44] Matsubara T, Sawano K (1995). Proteolytic cleavage of vitellogenin and yolk proteins during vitellogenin uptake and oocyte maturation in barfin flounder (*Verasper moseri*).. J Exp Zool.

[45] Tyler CR, Sumpter JP, Campbell PM (1991). Uptake of vitellogenin into oocytes during early vitellogenic development in the rainbow trout, *Oncorhynchus mykiss* (Walbaum).. J Fish Biol.

[46] Kambysellis MP (1977). Genetic and Hormonal Regulation of Vitellogenesis in Drosophila.. Amer Zool.

[47] Davis RE, Kelly TJ, Masler EP, Fescemyer HW, Thyagaraja BS (1990). Hormonal control of vitellogenesis in the gypsy moth, *Lymantria dispar* (L.): Suppression of haemolymph vitellogenin by the juvenile hormone analogue, methoprene.. J Insect Physiol.

[48] Tsutsui N, Kawazoe I, Ohira T, Jasmani S, Yang W-J (2000). Molecular characterization of a cDNA encoding vitellogenin and its expression in the hepatopancreas and ovary during vitellogenesis in the Kuruma prawn, *Penaeus japonicus*.. Zool Sci.

[49] Mak ASC, Choi CL, Tiu SHK, Hui JHL, He J-G (2005). Vitellogenesis in the red crab *Charybdis feriatus*: Hepatopancreas-specific expression and farnesoic acid stimulation of vitellogenin gene expression.. Mol Reprod Devel.

[50] Eckelbarger KJ (1986). Vitellogenic mechanisms and the allocation of energy to offspring in polychaetes.. Bull Mar Sci.

[51] Lucas MI, Walker G, Holland DL, Crisp DJ (1979). An energy budget for the free-swimming and metamorphosing larvae of *Balanus balanoides* (Crustacea: Cirripedia).. Mar Biol.

[52] Matsumura K, Mori S, Nagano M, Fusetani N (1998). Lentil lectin inhibits adult extract-induced settlement of the barnacle, *Balanus amphitrite*.. J Exp Zool.

[53] Jouuchi T, Satuito C, Kitamura H (2007). Sugar compound products of the periphytic diatom *Navicula ramosissima* induce larval settlement in the barnacle *Amphibalanus amphitrite*.. Mar Biol.

[54] Glenner H, Høeg JT (1995). Scanning electron microscopy of cypris larvae of *Balanus amphitrite* (Cirripedia: Thoracica: Balanomorpha).. J Crustacean Biol.

[55] Jensen PG, Moyse J, Høeg J, Al-Yahya H (1994). Comparative SEM studies of lattice organs: putative sensory structures on the carapace of larvae from ascothoracida and cirripedia (Crustacea Maxillopoda Thecostraca).. Acta Zool.

[56] Saroyan JR, Lindner E, Dooley CA (1970). Repair and reattachment in the balanidae as related to their cementing mechanism.. Biol Bull.

[57] Kamino K, Odo S, Maruyama T (1996). Cement proteins of the acorn barnacle, *Megabalanus rosa*.. Biol Bull.

[58] Kamino K, Inoue K, Maruyama T, Takamatsu N, Harayama S (2000). Barnacle cement proteins. Importance of disulfide bonds in their insolubility.. J Biol Chem.

[59] Van der Geer P, Hunter T, Lingdberg R (1994). Receptor protein-tyrosine kinases and their signal transduction pathways.. Annu Rev Cell Biol.

[60] Rewitz KF, Yamanaka N, Gilbert LI, O'Connor MB (2009). The insect neuropeptide PTTH activates receptor tyrosine kinase torso to initiate metamorphosis.. Science.

[61] Riddiford LM, Truman JW (1993). Hormone receptors and the regulation of insect metamorphosis.. Amer Zool.

[62] Wilcockson DC, Webster SG (2008). Identification and developmental expression of mRNAs encoding putative insect cuticle hardening hormone, bursicon in the green shore crab *Carcinus maenas*.. Gen Comp Endocrinol.

[63] Levine MZ, Harrison PJH, Walthall WW, Tai PC, Derby CD (2001). A CUB-serine protease in the olfactory organ of the spiny lobster *Panulirus argus*.. J Neurobiol.

[64] Harrison DCS (1999). Morphology of the nervous system of the barnacle cypris larva (*Balanus amphitrite* Darwin) revealed by light and electron microscopy.. Biol Bull.

[65] Wang H, Zhang H, Wong YH, Voolstra C, Ravasi T (2010). Rapid transcriptome and proteome profiling of a non-model marine invertebrate, *Bugula neritina*.. Proteomics.

[66] Wang L, Feng Z, Wang X, Zhang X (2010). DEGseq: An R package for identifying differentially expressed genes from RNA-seq data.. Bioinformatics.

[67] Livak KJ, Schmittgen TD (2001). Analysis of relative gene expression data using real-time quantitative PCR and the 2(-Delta Delta C(T)) method.. Methods.

[68] Thisse C, Thisse B (2008). High-resolution *in situ* hybridization to whole-mount zebrafish embryos.. Nat Protoc.

