# Supplementary material for: Toward an Understanding of the Molecular Mechanisms of Barnacle Larval Settlement: A Comparative Transcriptomic Approach
Source: PLoS One. 2011 Jul 29;6(7):e22913. doi: 10.1371/journal.pone.0022913 (PMC3146488; doi:10.1371/journal.pone.0022913)
Supplement: Text S1 — Method of putative bacterial gene identification. (DOC) [file pone.0022913.s001.doc]

**Text S1. Identification of putative bacterial genes.**

We combined nine bacterial genomes downloaded from NCBI to form a database to align the barnacle dataset against using the standalone BLASTn program (cutoff: e-value<1e-10). These nine bacterial genomes were: *Bacillus subtilis* 168 uid57675, *Bacillus subtilis* BSn5 uid62463, *Bacillus subtilis* spizizenii W23 uid51879, *Escherichia coli* 536 uid58531, *Escherichia coli* 55989 uid59383, *Escherichia coli* ATCC 8739 uid58783, *Pseudomonas aeruginosa* PA7 uid58627, *Pseudomonas aeruginosa* PAO1 uid57945, *Pseudomonas aeruginosa* UCBPP PA14 uid57977. There were 340 ESTs with significant hits in the bacterial genome database. These 340 sequences were then searched against NCBI NR peptide database using the BLASTx program to more accurately annotate these ESTs. The BLASTx results are shown in Table S3.
