# Supplementary material for: Toward an Understanding of the Molecular Mechanisms of Barnacle Larval Settlement: A Comparative Transcriptomic Approach
Source: PLoS One. 2011 Jul 29;6(7):e22913. doi: 10.1371/journal.pone.0022913 (PMC3146488; doi:10.1371/journal.pone.0022913)
Supplement: Table S4 — List of primers for genes under real-time PCR assay. (DOC) [file pone.0022913.s005.doc]

**Table S4. List of primers for genes under real-time PCR assay.**

| **Gene name** | **Gene’s symbol** | **Best match in NCBI** | **E value** | **EST number** | **Forward primer** | **Reverse primer** | **Amplicon length (bp)** |
| --- | --- | --- | --- | --- | --- | --- | --- |
| **Eph receptor tyrosine kinase** | *Ba-eph* | *Ixodes scapularis* (XP_002415239.1) | 5e-59 | isotig 14907 | AAGAGCATAGAGAAGGGATACCG | GAGACTGTCCAGCGTTTTGAC | 141 |
| **Tyrosine-protein kinase-like 7** | *Ba-ptk7* | *Apis mellifera*  (XP_394162.3) | 5e-49 | § | AGGTACCAGTGTGTGGTGAACA | AGTACAGCCGACCCGTCTTA | 145 |
| **Discoidin domain receptor** | *Ba-ddr1* | *Pediculus*  *humanus corporis* (XP_002431712.1) | 2e-65 | isotig 13163 | TGCTCACCTACGCTCGACAGCA | TCTCTCGGACAATCGTGCGGCT | 130 |
| **Discoidin domain receptor** | *Ba-ddr2* | *Drosophila yakuba* (XP_002088205.1) | 1e-20 | isotig 13349 | CAGCACTCGCACATCAGGTCGA | TGGTCGTTCTCGGTGACGCTCT | 188 |
| **Mannose receptor** | *Ba-mnr1* | *Saccoglossus kowalevskii* (XP_002742001.1) | 8e-18 | isotig 06254 | ATGGCTGGACAAGCGTTGGCTC | TCGTAGTCGGACACTGCTGCCA | 124 |
| **Mannose receptor** | *Ba-mnr2* | *Gallus gallus* (XP_418617.2) | 9e-17 | isotig 10262 | TCCTCACTTGTCACCACCGCCT | TCGGCGTCGAGTGAAGACCGAT | 115 |
| **Mannose receptor** | *Ba-mnr3* | *Taeniopygia guttata* (XP_00219262.1) | 2e-19 | isotig 10448 | TACCGACTGAGCAGCTATGAGA | TGTCAATGATGGACTTCTGCTT | 139 |
| **Mannose receptor** | *Ba-mnr4* | *Saccoglossus kowalevskii* (XP_002742001.1) | 2e-13 | isotig 16920 | TCGATTATACCAACTGGGAGGT | CAGTCTCGCAGACATAGGACAG | 171 |
| **Cubilin** | *Ba-cubn1* | *Panulirus argus* (AAK48894.1) | 1e-17 | isotig 07947 | TGGCGAGGCCTATGGCAAAGTC | TGTCCGGGTAGTTGGGCGAAGT | 146 |
| **Cubilin** | *Ba-cubn2* | *Ciona intestinalis* (XP_002128933.1) | 1e-13 | isotig 08999 | TGGCCAGCGATCCGTCAAACAC | TGTTGTTGCAGTGGCGGACACC | 120 |
| **Cubilin** | *Ba-cubn3* | *Ciona intestinalis* (XP_002129050.1) | 4e-06 | isotig 07962 | TCAGCAGCATGACGTTCGCCTG | AGTAGAAGCCGGGTCCCGTGAT | 186 |
| **20 kDa-cement protein homologue** | *Ba-cph1* | *Balanus albicostatus* (BAF96022.1) | 2e-09 | isotig 06908 | CAACTTCAACTGCACGGATAGT | AGCTAGGCTCGGTCTCGTTG | 104 |
| **20 kDa-cement protein homologue** | *Ba-cph2* | *Balanus albicostatus* (BAF96022.1) | 4e-19 | isotig 16872 | ACAACTTGCTTTGTGTCTTACCG | ACGTGTCTTGACAGTGGAAGC | 116 |
| **Bursicon-like** | *Ba-bursl* | *Acyrthosiphon pisum* (XP_001946341.1) | 1e-46 | F6924YJ02D1UMQ | AAAAGTTCCGAAAGATCGTGAC | ATCGACTCGAGCGACATACC | 134 |
| **Ecdysone-inducible receptor** | *Ba-eir* | *Blattella germanica* (CAM97373.1) | 3e-71 | isotig 08528 | ATCCAGCAGAAGATCCAGTACAA | ATACATTTCTTGAGCCGGCAGTA | 104 |
| **Ecdysone receptor** | *Ba-ecr1* | *Manduca sexta* (P49883.1) | 3e-26 | isotig 10717 | GCCGAAGTATGACGAAGAACAT | AGACACTTCTTCAGGCGACATT | 109 |
| **Ecdysone receptor** | *Ba-ecr2* | *Blattella germanica* (CAJ01677.1) | 8e-13 | isotig 10718 | ACCGCCATCGACATCTTCTC | AAGTCCACGTACGCCCTCAG | 104 |
| **Vitellogenin** | *Ba-vtg* | *Lepeophtheirus salmonis* (ABU41134.1) | 3e-09 | isotig 06660 | ACTGGCCTCGACTACCACCT | TCTCCAGTCCACTTCAGGATG | 132 |

§ The sequence of Tyrosine-protein kinase-like 7 gene (*ptk7*) was aligned from three singlets, F51RSDG03HDSIL, GBQDZ6L01BDPXW and GBQDZ6L01DI8ET manually. The identity of the overlapped nucleotide sequences was over 97%.
