# Supplementary material for: Toward an Understanding of the Molecular Mechanisms of Barnacle Larval Settlement: A Comparative Transcriptomic Approach
Source: PLoS One. 2011 Jul 29;6(7):e22913. doi: 10.1371/journal.pone.0022913 (PMC3146488; doi:10.1371/journal.pone.0022913)
Supplement: Table S5 — List of primers for genes under ISH study. (DOC) [file pone.0022913.s006.doc]

**Table S5. List of primers for genes under ISH study.**

| **Gene** | **Gene’s symbol** | **EST number** | **Forward primer** | **Reverse primer** | **Amplicon length (bp)** |
| --- | --- | --- | --- | --- | --- |
| **Mannose receptor** | *mnr1* | isotig 06254 | GATTCGTCGTCGCCATGGTCGT | TCGTCTGCCTCCAGGCCGTTTA | 901 |
| **Mannose receptor** | *mnr2* | isotig 10262 | AAGCTGAGCGACACGACGCAGA | ACACAGTCGTTCCCAGGTCCGA | 719 |
| **20kDa-cement protein homologue** | *cph1* | isotig 06908 | CAACTGCACGGATAGTTGTGACT | AATTGAGTCGCAGTCGCAGT | 202 |
| **20kDa-cement protein homologue** | *cph2* | isotig 16872 | GTAATAGTGGCTGTTTTCGTTGC | GATGCTGCATTCAATCGAGTC | 300 |
